# Trauma-associated growth of suspected dormant micrometastasis

**DOI:** 10.1186/1471-2407-5-94

**Published:** 2005-08-04

**Authors:** Nagi S El Saghir, Ihab I Elhajj, Fady B Geara, Mukbil H Hourani

**Affiliations:** 1 Department of Internal Medicine, American University of Beirut Medical Center, Beirut, Lebanon; 2 Department of Radiation Therapy, American University of Beirut Medical Center, Beirut, Lebanon; 3 Department of Diagnostic Radiology, American University of Beirut Medical Center, Beirut, Lebanon

## Abstract

**Background:**

Cancer patients may harbor micrometastases that remain dormant, clinically undetectable during a variable period of time. A traumatic event or surgery may trigger the balance towards tumor growth as a result of associated angiogenesis, cytokine and growth factors release.

**Case presentation:**

We describe a patient with non-small lung cancer who had a rapid tumor growth and recurrence at a minor trauma site of his skull bone.

**Conclusion:**

This case is an illustration of the phenomenon of tumor growth after trauma or surgery and its associated cellular mechanisms. This phenomenon deserves further investigation and study.

## Background

Cancer patients may harbor micrometastases which are responsible for recurrent disease. Micrometastases remain dormant as a result of a balance between tumor cell proliferation and an equivalent rate of cell death [1,2]. Surgical interventions may trigger tumor growth an effect associated with angiogenesis, cytokines and growth factors release [3]. We report a patient with non-small lung cancer who had a rapid tumor growth and recurrence at a minor trauma site of his parietal skull bone. We suggest that the phenomenon of tumor growth after trauma or surgery deserves further investigation and study.

## Case presentation

A 43 year-old smoker was diagnosed with a non small-cell lung cancer (NSCLC) in December 2001. He had a left upper lobe mass with adenopathy in the aorto-pulmonary window. CT-guided FNA of the lung lesion revealed a poorly differentiated NSCLC. Metastatic work-up showed only a suspicious 4 × 2 cm right supra-acetabular lesion of which a core biopsy was negative for malignancy. His total body bone scan showed no abnormal uptake in the skull and CT of brain was negative for metastatic disease.

**Figure 1:**
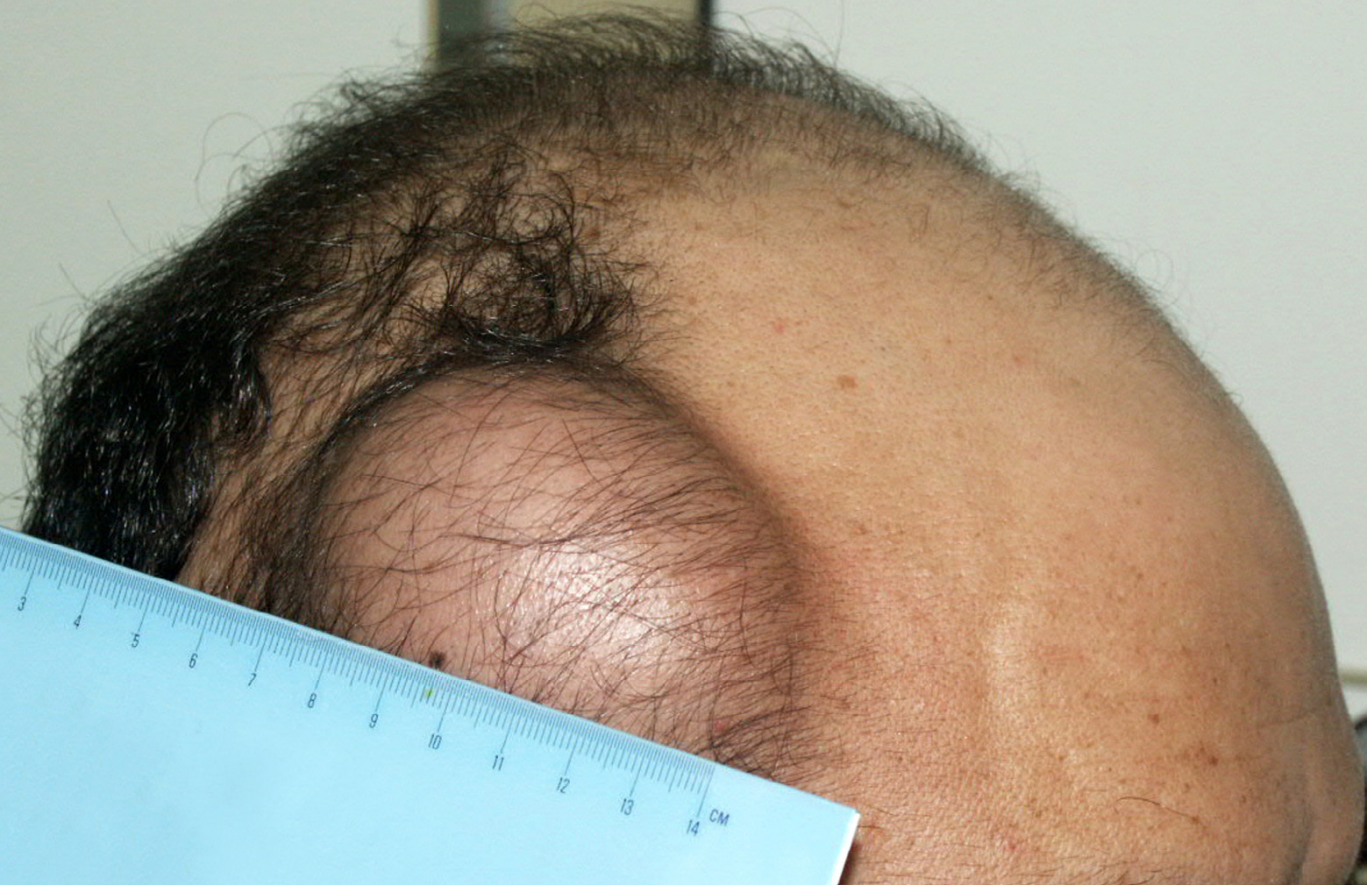
Photograph of the patient showing the described 7 cm mass at the site of the trauma at the right lateral side of his head.

**Figure 2:**
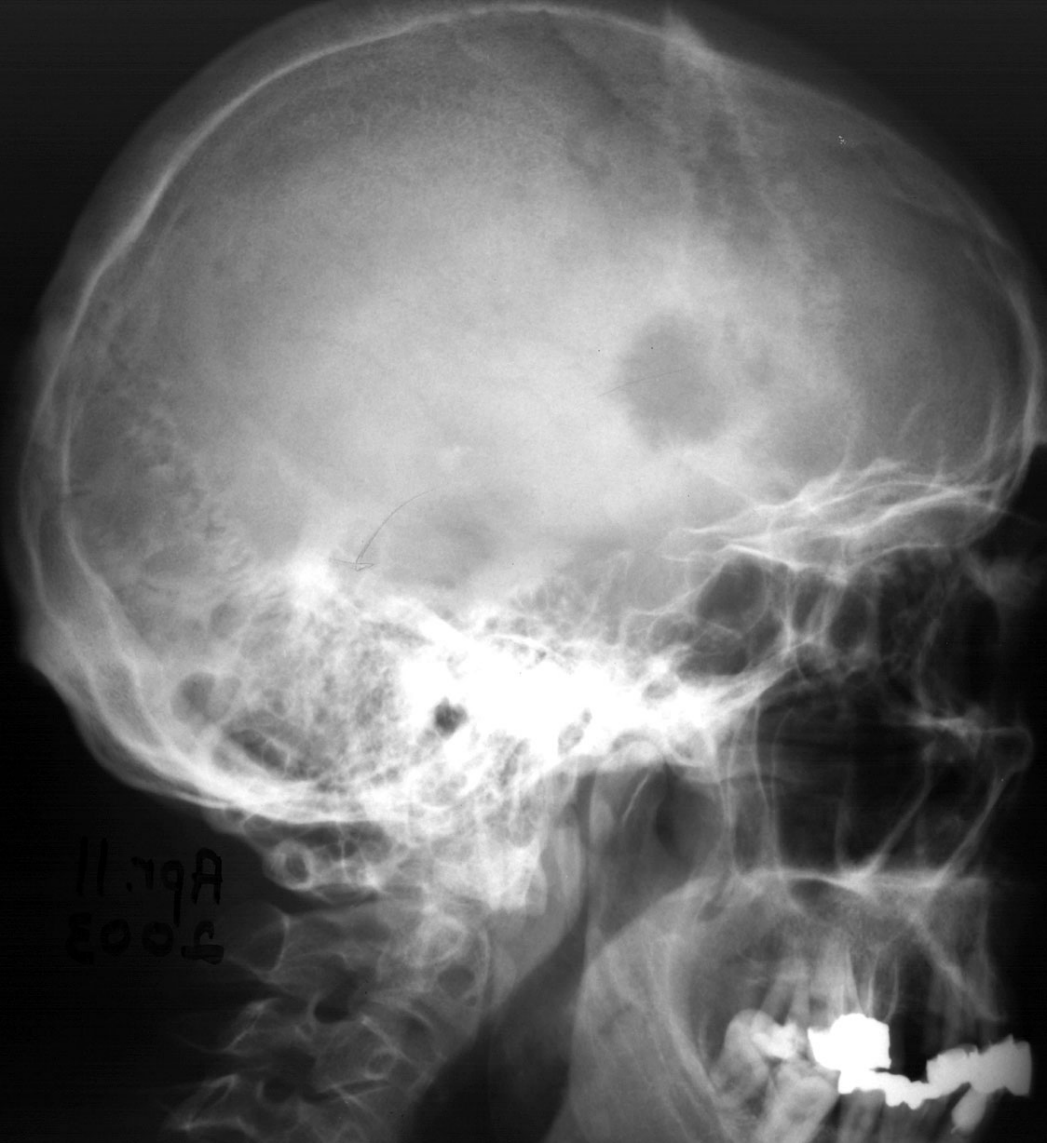
Plain skull x-ray film showing an irregular lytic lesion of the right parietal lobe.

**Figure 3:**
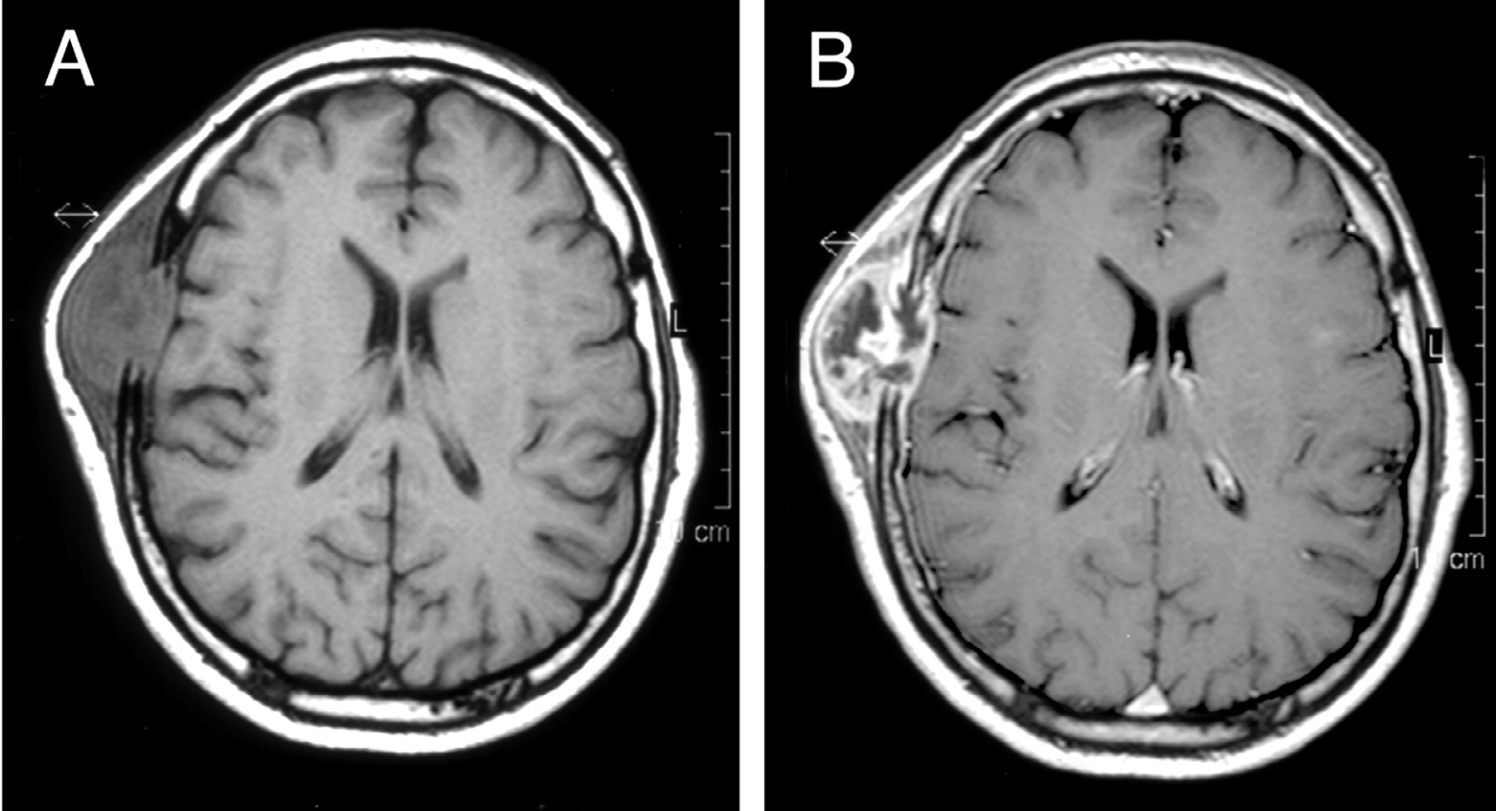
T1 weighted MRI of brain before (figure 3A) and after Gadolinium contrast enhancement (figure 3B>), showing a metastatic deposit involving the right frontal bone with a large extracranial soft tissue component and meningeal invasion.

He was given chemotherapy and radiotherapy and had a very good response. In April 2003, the patient reported that he suffered a minor trauma to the right lateral side of his head when he was in the passenger front seat of his car that rolled over a road bump. He reported a new small swelling that grew rapidly over a one month period. On physical examination, the mass was rubbery and not freely mobile measuring 7 cm in largest dimension (Figure 1). Plain skull x-ray films showed a 2 cm irregular lytic lesion of the right parietal bone (Figure 2). There were no underlying bone fractures. T1 weighted MRI of brain before (Figure 3A) and after Gadolinium contrast enhancement (Figure 3B), showed a metastatic deposit involving the right parietal bone with a large extracranial soft tissue component and meningeal invasion. T2 weighted image demonstrated the above described findings with meningeal thickening. There was no evidence of metastatic brain disease. Bone window of a non-enhanced CT of brain for radiation therapy planning purposes, showed a large lytic lesion with a soft tissue component of the right parietal bone. Chest X-ray showed no change in the residual ill defined left upper lobe density that the patient had in 2002. We concluded that the patient had an isolated tumor recurrence in his skull and proceeded with radiation therapy. The lesion showed decrease in size by the end of radiation. Fifteen days later, the family reported that the patient died at home of massive hemoptysis.

## Discussion

We report the case of a patient with NSCLC who had an unusual recurrence in his skull after a direct minor trauma. The traumatic event was followed by the development of a small swelling that grew rapidly and manifested itself as an unusual metastatic lesion in the parietal skull bone as demonstrated in the accompanying figures. This event may be explained by the presence of dormant cancer cells might have been stimulated by a new environment of stimulatory factors. Several authors have investigated similar issues and incriminated growth factors, cytokines and angiogenic mechanisms [1]. It is now well known that cancer patients may harbor micrometastases and dormant cancer cells. Metastases remain dormant and clinically undetectable during a variable period of time when tumor cell proliferation is balanced by an equivalent rate of cell death.

Establishment and growth of metastases are thought to be influenced by endogenous inhibitors of angiogenesis which keep metastases in a non-proliferating quiescent state characterized by normal proliferation, increased apoptosis, and insufficient neovascularization [2,4]. Folkman suggested that, for tumors to grow and develop metastatic potential, they must make an "angiogenic switch" through perturbing the local balance of proangiogenic and antiangiogenic factors [5]. Lesions in the nervous system induce rapid activation of glial cells and under certain conditions additional recruitment of granulocytes, T-cells and monocytes/macrophages from the blood stream triggered by upregulation of cell adhesions molecules, chemokines, and cytokines [6].

Cytokines and chemokines may act to promote tumors by several mechanisms that include: DNA damage, bypass of p53, angiogenesis, growth stimulation, enhanced survival, subversion of immunity and enhanced invasion [7]. Some chemokines (eg, IL-8) are proangiogenic whereas others such as IP-10 have antiangiogenic activity. Chemokines have direct actions on microvacsular endothelial cells. In addition, CC chemokines may inhibit or stimulate angiogenesis indirectly, via their influence on tumor-associated macrophages. Inflammatory macrophages produce transforming growth factor β1 (TGF-β1) that is itself angiogenic and induces production of vascular endothelial growth factor (VEGF) [8]. This activation causes temporarily dormant micrometatases to vascularize, and thus to enter a rapid growth phase. In many tumors (eg, NSCLC and pancreatic carcinoma) it is postulated that the balance between proangiogenic and antiangiogenic cytokines and chemokines, rather than absolute amounts, regulates tumor angiogenesis [1].

A traumatic event triggers several mechanisms of soft tissue and bone repair of which angiogenesis is part. Dormant cancer cells at the site of tissue trauma and thereby exposed to pro-inflammatory mediators, may be sufficiently stimulated to overcome dormancy. Lee et al. [9] studied the effect of trauma on the implantation of metastatic tumor in bone in mice. The results suggest that the healing wound is a privileged site for experimental metastasis, particularly in the early stages. It is likely that the proteins in the blood clotting cascade are involved in local tumor implantation [10].

Another mechanism whereby trauma may alter subsequent tumor growth is that cytokine genes are highly polymorphic and since polymorphisms are frequently in regions of DNA that regulate transcription or post-transcriptional events, the cytokines may be cancer-modified genes [11].

Baum suggested that the act of surgery can provoke the outgrowth of dormant micrometatstasis [12,13]. Coffey et al. [3] reviewed mechanisms by which tumor excision may alter residual tumor growth and discussed the potential use of peri-operative chemotherapy, antiendotoxin agents, immunotherapy and biomodulation with use of dendritic-cell vaccines. Retsky et al. [14] hypothesized that induced angiogenesis after surgery in premenopausal node-positive breast cancer patients is a major underlying reason why adjuvant chemotherapy works particularly well for those patients. Our present case is an illustration of cellular mechanisms also associated with trauma that might either stimulate tumor growth of already present and dormant cells, or attract and trap circulating tumor cells.

## Conclusion

Most traumatic and surgical events in cancer patients do not lead to tumor growth nor metastases, and swellings occurring after trauma do not require biopsy to prove their nature. However, we suggest that the phenomenon of tumor growth after trauma or surgery deserves further investigation and study. Antiangiogenic drugs might have a potential role for investigation in some circumstances of cancer patients in remission who require surgery, or are subject to a traumatic event.

## Competing interests

The author(s) declare that they have no competing interests.

## Authors' contributions

NSES, the corresponding author, was the main operator in charge of the case, the idea and the manuscript. IIEH assisted in the literature search and manuscript. FBG was involved in the patient's management and radiation therapy. MHH was involved in imaging and preparation of figures. All authors contributed to the preparation of the manuscript. All authors read and approved the final version of the manuscript.

## Pre-publication history

The pre-publication history for this paper can be accessed here:



## References

[B1] O'Byrne KJ, Dalgleish AG, Browning MJ, Steward WP, Harris AL (2000). The relationship between angiogenesis and the immune response in carcinogenesis and the progression of malignant disease. Eur J Cancer.

[B2] Holmgren L, O'Reilly MS, Folkman J (1995). Dormancy of micrometastases: balanced proliferation and apoptosis in the presence of angiogenesis suppression. Nat Med.

[B3] Coffey JC, Wang JH, Smith MJ, Bouchier-Hayes D, Cotter TG, Redmond HP (2003). Excisional surgery for cancer cure: therapy at a cost. Lancet Oncol.

[B4] Kirsch M, Schackert G, Black PM (2004). Metastasis and angiogenesis. Cancer Treat Res.

[B5] Folkman J (2002). Role of angiogenesis in tumor growth and metastasis. Semin Oncol.

[B6] Stoll G, Jander S, Schroeter M (2002). Detrimental and beneficial effects of injury-induced inflammation and cytokine expression in the nervous system. Adv Exp Med Biol.

[B7] Balkwill F, Mantovani A (2001). Inflammation and cancer: back to Virchow?. Lancet.

[B8] Opdenakker G, Van Damme J (1992). Chemotactic factors, passive invasion and metastasis of cancer cells. Immunol Today.

[B9] Lee JY, Murphy SM, Scanlon EF (1994). Effect of trauma on implantation of metastatic tumor in bone in mice. J Surg Oncol.

[B10] Rosenberg GA (1995). Matrix metalloproteinases in brain injury. J Neurotrauma.

[B11] Jaiswal M, LaRusso NF, Burgart LJ, Gores GJ (2000). Inflammatory cytokines induce DNA damage and inhibit DNA repair in cholangiocarcinoma cells by a nitric oxide-dependent mechanism. Cancer Res.

[B12] Baum M (1996). Does surgery disseminate or accelerate cancer?. Lancet.

[B13] Baum M (2004). Does the act of surgery provoke activation of "latent" metastases in early breast cancer?. Breast Cancer Res.

[B14] Retsky M, Bonadonna G, Demicheli R, Folkman J, Hrushesky W, Valagussa P (2004). Hypothesis: Induced angiogenesis after surgery in premenopausal node-positive breast cancer patients is a major underlying reason why adjuvant chemotherapy works particularly well for those patients. Breast Cancer Res.

